# Punicalagin and Ellagic Acid Demonstrate Antimutagenic Activity and Inhibition of Benzo[a]pyrene Induced DNA Adducts

**DOI:** 10.1155/2014/467465

**Published:** 2014-05-14

**Authors:** Maryam Zahin, Iqbal Ahmad, Ramesh C. Gupta, Farrukh Aqil

**Affiliations:** ^1^Department of Agricultural Microbiology, Aligarh Muslim University, Aligarh 202002, India; ^2^James Graham Brown Cancer Center, University of Louisville, Louisville, KY 40202, USA; ^3^Department of Pharmacology and Toxicology, University of Louisville, Louisville, KY 40202, USA; ^4^Department of Medicine, University of Louisville, Delia Baxter II, Room 304B, 580 S. Preston Street, Louisville, KY 40202, USA

## Abstract

Punicalagin (PC) is an ellagitannin found in the fruit peel of *Punica granatum*. We have demonstrated antioxidant and antigenotoxic properties of *Punica granatum* and showed that PC and ellagic acid (EA) are its major constituents. In this study, we demonstrate the antimutagenic potential, inhibition of BP-induced DNA damage, and antiproliferative activity of PC and EA. Incubation of BP with rat liver microsomes, appropriate cofactors, and DNA in the presence of vehicle or PC and EA showed significant inhibition of the resultant DNA adducts, with essentially complete inhibition (97%) at 40 **μ**M by PC and 77% inhibition by EA. Antimutagenicity was tested by Ames test. PC and EA dose-dependently and markedly antagonized the effect of tested mutagens, sodium azide, methyl methanesulfonate, benzo[a]pyrene, and 2-aminoflourine, with maximum inhibition of mutagenicity up to 90 percent. Almost all the doses tested (50–500 **μ**M) exhibited significant antimutagenicity. A profound antiproliferative effect on human lung cancer cells was also shown with PC and EA. Together, our data show that PC and EA are pomegranate bioactives responsible for inhibition of BP-induced DNA adducts and strong antimutagenic, antiproliferative activities. However, these compounds are to be evaluated in suitable animal model to assess their therapeutic efficacy against cancer.

## 1. Introduction

Over the past few decades, tremendous outcomes have been resulted by exploring antioxidant and antimutagenic potential of medicinal plants. It is widely accepted that oxidative modification of DNA, protein, lipids, and small cellular molecules by both exogenous and endogenous reactive oxygen species including free radicals and nonfree radicals plays an important role in a wide range of common diseases including cancer and age related degenerative diseases [1, 2]. The human body possesses innate defence mechanisms to counter free radicals. Plant secondary metabolites such as phenolics, flavonoids, and terpenoids play an important role in the defence against free radicals [3]. Moreover, these natural antioxidants may reduce or inhibit the mutagenic potential of mutagens, promutagens, and carcinogens [4, 5]. Therefore, the discovery and the exploration of compounds possessing antioxidant, antimutagenic, and anticancer properties are now fetching great practical and therapeutic significance.

The formation of DNA adducts (i.e., carcinogens covalently bound to DNA) is widely considered a prerequisite for the initiation and progression of cancer development. Many carcinogens are known to induce the formation of DNA adducts [6] and the presence of DNA adducts in humans has been strongly correlated with an increased risk of cancer development [7]. For example, human studies have shown a higher accumulation of tissue DNA adducts in cigarette smokers than in nonsmokers or individuals who have never smoked, indicating that DNA adduct formation is a viable target for the treatment of cancer [8].

Benzo[a]pyrene (BP) is one of the most potent and extensively studied carcinogens. In a cellular system, BP is metabolized to the electrophilic metabolite, benzo[a]pyrene-7,8-diol-9,10-epoxide (BPDE), that attaches covalently to DNA bases, primarily deoxyguanosine. Inflammatory response to chemical carcinogens and formation of DNA adducts are generally considered a prerequisite in the process of chemical carcinogenesis [9]. Accumulation of DNA adducts resulting from chronic exposure to low-level environmental carcinogens has been used as a possible measure of exposure to carcinogens and cancer risk assessment [10].

Natural antimutagens from edible and medicinal plants are of particular importance because they may be useful for inhibition of DNA adducts leading to human cancer prevention and have no undesirable xenobiotic effects on living organisms [11, 12]. Encouraging reports on antimutagenic properties of edible plants have led to increase interest in search of natural phytoantimutagens from medicinal plants [13, 14]. Among them is* Punica granatum* (pomegranate), which have been used widely as antimicrobial, antioxidant, antimutagenic, and anticancer [15–17]. Pomegranate has been shown to possess high amount of ellagitannins (ETs) such as punicalagin (PC), punicalin, gallagic acid, ellagic acid (EA), and EA-glycosides [18, 19].

**Figure 1:**
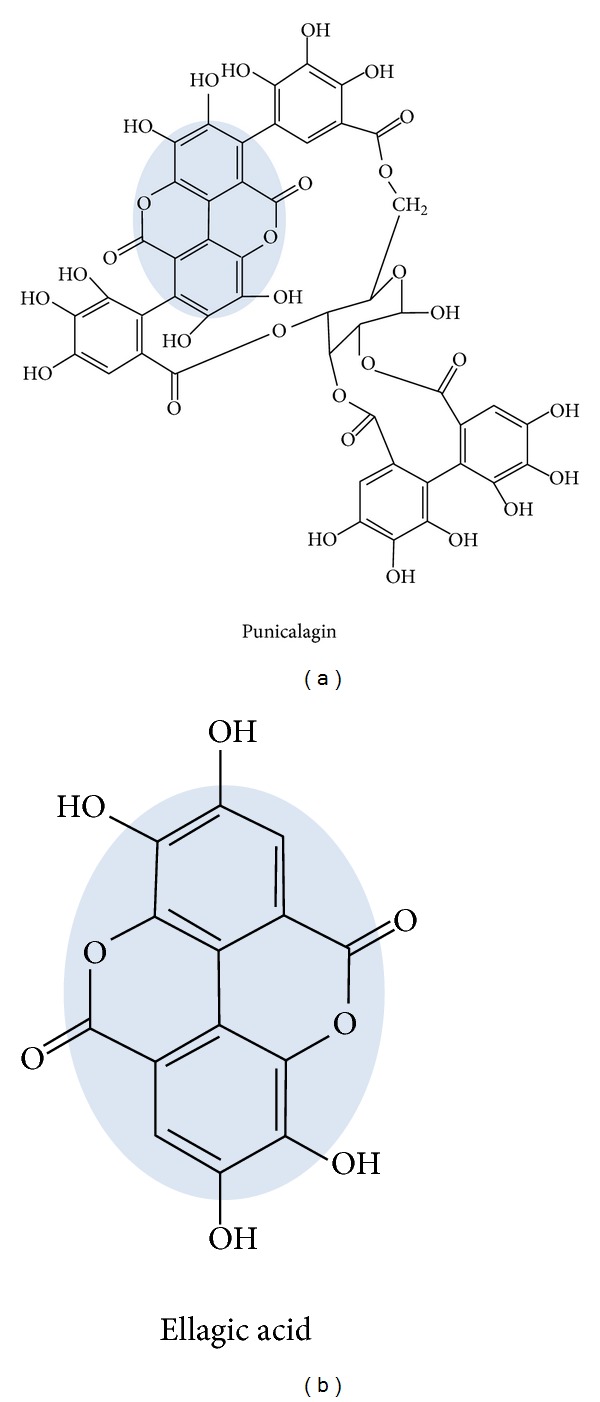
Chemical structures of punicalagin and ellagic acid.

Punicalagin and ellagic acid (Figure 1) emerged out to be the most elaborated groups of compounds, known for their potential role in various biological activities. Like other polyphenols, PC, EA, and their derived metabolites possess a wide range of biological activities, which suggested that they could have beneficial effects on human health. PC and EA have antioxidant functions and possess strong anti-inflammatory, antiproliferative, hepatoprotective, and antigenotoxic properties [20–23].

PC and EA also exhibit anticancer properties* in vitro* and* in vivo* [17, 24]. However, studies on antimutagenic potential on these compounds are scanty. Therefore, considering our results and previous findings by other workers, we extended our study to isolate the key compounds, PC from* P. granatum* peel extracts. In this study we demonstrate antimutagenic properties of PC and EA against the mutagenicity induced by mutagens (sodium azide and methyl methanesulfonate) and promutagens (BP and 2AF) in Ames* Salmonella* assay. This study, to the best of our knowledge, is the first to show antimutagenic properties against a panel of mutagens/carcinogens and procarcinogens. We also examined protective effect of PC and EA against BP-induced DNA adducts and antiproliferative activity against lung cancer cells* in vitro*.

## 2. Materials and Methods

### 2.1. Bacterial Strains and Chemicals

The* Salmonella typhimurium* strains TA97a, TA98, TA100, and TA102 were kindly provided by Prof. B. N. Ames, University of California, Berkeley, USA. The details of the strains are provided in the Supplementary Table S1 (see Supplementary Material available online at http://dx.doi.org/10.1155/2014/467465). Sodium azide (NaN_3_) was purchased from HiMedia Lab. (Mumbai, India). D-glucose-6-phosphate disodium salt, nicotinamide adenine dinucleotide phosphate sodium salt, sodium phosphate, ammonium molybdate, neocuproine, L-histidine monohydrate, D-biotin, 2-aminofluorene (2AF), benzo[a]pyrene, and ellagic acid were purchased from Sigma-Aldrich (St. Louis, MO, USA). Methyl methanesulfonate (MMS) and trichloroacetic acid were purchased from Sisco Research Laboratories Pvt. Ltd., Mumbai, India. All other reagents used to prepare buffers and media were of analytical grade.

### 2.2. Preparation of the Extract and Isolation of Punicalagin

*Punica granatum* (pomegranate) fruits peel extracts (30% enriched for punicalagins) were purchased from Pharmanza Inc. (Gujarat, India). The extracts were prepared by dissolving 10 g of peel powder in 5 vol (50 mL) of water. Samples were then centrifuged at 6000 g for 10 min and decanted and pooled extracts from three extractions were dried under reduced pressure using Rota-vapor at 45°C. PC was isolated by Amberlite XAD-16 and C18 column chromatography as described [19]. Isolated PC was at least 97% pure and essentially free of EA as determined by HPLC-UV.

### 2.3. Antimutagenicity Assay

The* Salmonella* histidine point mutation assay described by Maron and Ames [25] was used to test the antimutagenic activity of PC and EA as described earlier [13, 26]. In the preincubation experiment, test compounds and mutagen, each having a volume of 0.1 mL of varying concentrations, were preincubated at 37°C for 30 min and then 0.1 mL of 1 × 10^7^ CFU/mL density of the bacterial culture was added, followed by the addition of 2.5 mL of top agar at 45°C (containing 0.5% NaCl and 0.6% agar) supplemented with 0.5 mM histidine-biotin. The influence of metabolic activation of promutagens, BP and 2AF was tested using 500 *μ*L of S9 mixture (0.04 mg proteins/mL of mix). The S9 microsome fraction was prepared from the livers of rats treated with Aroclor 1254 using standard protocols [27]. The combined solutions were vortexed and poured onto minimal glucose plates (40% glucose solution and Vogel Bonner medium). The plates were incubated at 37°C for 48 h and the numbers of histidine-independent revertants colonies were scored.

Survival of the bacteria was routinely monitored for each experiment. Parallel controls were run with compounds alone at all concentrations to test the possible toxicity. The concentrations of the test samples for investigating the antimutagenicity were 50, 100, 200, and 500 *μ*M. PC and EA were tested against mutagens sodium azide (1.5 *μ*g/0.1 mL/plate) and MMS (1 *μ*g/0.1 mL/plate) as well as against promutagens, BP and 2AF in TA97a and TA98 (frame shift mutation), TA100 (base pair substitution), and TA102 (transition mutation) tester strains (Supplementary Table S1). All the test samples and mutagens were dissolved in DMSO (final conc., 0.01%). In each case, there was no toxicity observed and the numbers of spontaneous revertants were identical with the DMSO vehicle control. Non-toxic concentrations were categorized as those where there was a well-developed lawn, almost similar size of colonies, and no statistical difference in the number of spontaneous revertants in test and control plates. Plates were set up in triplicate for each concentration and the entire experiment was repeated three times. Inhibition of mutagenicity was expressed as percentage decrease of reverse mutation and calculated as

(1) $$\begin{array}{c} \text{Percent}\;\text{inhibition}=\left[\frac{\left(a-b\right)}{\left(a-c\right)}\right]\times 100, \end{array}$$

where *a* = number of histidine revertants induced by mutagen, *b* = number of histidine revertants induced by mutagen in the presence of test compound, and *c* = number of revertants induced in negative control.

### 2.4. Microsomal BP-DNA Adducts

*st*-DNA (300 *μ*g/mL) was preincubated with 50 mM Tris-HCl (pH 7.5), 1 mM MgCl_2_, 2.5 mM glucose-6-phosphate, 1 U/mL G6PDH, 0.5 mM NADP+, and *α*-naphthoflavone-induced rat liver microsomal proteins (1 mg/mL) in 1 mL for 10 min, in the presence of vehicle alone and PC and EA at 20 and 40 *μ*M. BP dissolved in DMSO was added at a final concentration of 1 *μ*M. The incubation was continued for another 30 min at 37°C and then reaction was terminated by the addition of EDTA and centrifugation (9,000 g; 10 min). DNA was isolated from the supernatant by removal of RNA and proteins by digestions with RNases A and T1 and proteinase K and a series of extractions with phenol, phenol: Sevag (chloroform : isoamyl alcohol, 24 : 1), and Sevag, followed by precipitation of the DNA with ethanol [29]. The DNA concentration was estimated spectrophotometrically.

### 2.5. Analysis of DNA Adducts

DNA adducts were analyzed by ^32^P-postlabeling as described earlier [29]. Briefly, 10 *μ*g of DNA was digested with micrococcal nuclease and spleen phosphodiesterase (MN/SPD). Before further treatment with nuclease P1 to enrich DNA adducts, an aliquot was removed for evaluation of normal nucleotide levels. DNA adducts and normal nucleotides were labelled with [*γ*-^32^P]ATP and T4 polynucleotide kinase. Labelled adducts were separated by multidirectional polyethyleneimine (PEI)-cellulose TLC using the following solvents: D1, 1.0 M sodium phosphate, pH 6.0; D3, 4 M lithium formate/7 M urea, pH 3.5; D4, 4 M ammonium hydroxide/isopropanol (1 : 0.9); and D5, 1.7 M sodium phosphate, pH 6.0. Normal nucleotides were resolved in 180 mM sodium phosphate, pH 6.0, by one-directional PEI-cellulose TLC. DNA adducts and normal nucleotides were detected and quantified by Packard InstantImager.

### 2.6. Cell Proliferation Assay and Measurements of Cell Viability

Inhibition of cell proliferation by PC and EA was measured with the MTT assay. Human lung cancer A549 and H1299 cells were obtained from ATCC (Manassas, VA, USA) and maintained in DMEM supplemented with 10% fetal calf serum (FCS), 1% penicillin/streptomycin. Cells were plated in 96-well culture plates (5 × 10^3^ cells/well). After 24 h incubation, cells were treated with vehicle alone (0.1% DMSO) and PC and EA (12.5–200 *μ*g/mL) extracts for 48 h. Then, the culture medium was replaced by 100 *μ*L of fresh medium containing 0.5 mg/mL MTT, and the plates were incubated for 2 h at 37°C. The medium was then removed and was replaced by 200 *μ*L of DMSO to solubilize the converted purple dye. The absorbance was measured with a spectrophotometer microplate reader at a wavelength of 570 nm.

### 2.7. Statistical Analysis

The results are presented as the average and standard error of three experiments with triplicate plates/dose/experiment. The regression analysis was carried out in Microsoft Excel 2007 between percent inhibition of mutagenicity and log values of concentrations of the plant extract.

## 3. Results 

Natural products have attracted much attention with respect to their benefits to human health and protective effects in various diseases including cancer [30]. We have previously demonstrated the antimicrobial [16], antioxidant, and antimutagenic potential and phytochemical analysis of* Punica granatum *[15, 19]. In this study we demonstrate the inhibition of BP-induced DNA adducts and antimutagenic and antiproliferative activities of PC and EA, the key components of pomegranate.

### 3.1. Evaluation of Mutagenicity of Tested Compounds

The mutagenicity and antimutagenicity of a compound can be detected using Ames test using specific indicator strains of* Salmonella typhimurium* [25]. No toxicity of PC and EA was found at tested 50–500 *μ*M concentrations as depicted in Tables 1–8 when tested in the absence of S9 fraction in Ames* Salmonella typhimurium* strains. However, at few concentrations there was slight but insignificant increase in the His^+^ revertants compared to spontaneous. No mutagenic activity of either of the compounds, PC or EA, was detected when investigated on any of* Salmonella* tester strains, TA97a, TA98, TA100, and TA102 either with or without S9 activation by plate incorporation assay (Tables 1–8).

### 3.2. PC and EA Are Highly Antimutagenic

The antimutagenic potential of PC and EA was evaluated using Ames* Salmonella* tester strains against direct acting mutagens (NaN_3_ and MMS), in the absence of S9, as well as against promutagens (BP and 2AF) with Aroclor induced rat liver S9. In the absence of test compounds these mutagens induced His^+^ revertants.

We have previously demonstrated that the methanol extract of* Punica granatum* has very high antimutagenic potential and contains PC and EA in addition to other trace compounds [15]. To evaluate the active principle, we further tested, in this study, PC and EA for antimutagenic potential at 50, 100, 250, and 500 *μ*M concentrations. The tested concentrations by plate-incorporation assay showed no sign of toxicity and mutagenicity to* Salmonella typhimurium *strains, either alone or in the presence of S9 mix.

BP- and 2AF-induced high number of His^+^ revertants in tester strains. PC showed a significant (*P* < 0.005) inhibition of BP- and 2AF-induced mutagenicity tested in the presence of S9 mix. At 500 *μ*M concentration, PC inhibited 2AF- and BP-induced mutagenicity in the range of 76.7% to 85.0% (Tables 1 and 2). The effect of PC was dose dependent as determined by regression analysis with the *R* ^2^ values ranging between 0.91 and 0.99.

Similarly, PC at the highest tested concentration (500 *μ*M) showed significant antimutagenicity against NaN_3_ and MMS. It inhibited sodium azide induced mutagenicity by 74.4% in TA97a followed by TA100 (74.3%), TA98 (65.3%), and TA102 (59.8%) strains as depicted in Table 3. MMS (1 *μ*g), when incubated with TA97a, increased the His^+^ revertants from 142 to 449 and it was almost completely reduced to 251 (an inhibition of 72%) by PC at 500 *μ*M (50 *μ*g/plate). The effect was similar in TA98, TA100, and TA102 to the inhibition of mutagenicity by 71%, 66%, and 75%, respectively (Table 4).

The antimutagenicity of EA against promutagens BP- and 2AF-induced mutagenicity was also highly significant (*P* < 0.005) as presented in Tables 5 and 6. EA showed dose dependent antimutagenic behavior against both BP and 2AF and reduced His^+^ revertants by 78.6% to 88.9%, respectively (Tables 5 and 6). The activity was dose dependent as determined by regression analysis between EA dose and antimutagenic response with *R* ^2^ values ranging between 0.93 and 0.99.

EA at a dose of 500 *μ*M showed significant (*P* < 0.005) antimutagenic activity against TA97a with a decrease in mutagenicity by 72.1% followed by TA100 (65.9%), TA98 (64.2%), and TA102 (62.3%) against NaN_3_ induced mutagenicity (Tables 7 and 8). A similar trend of activity was obtained against MMS-induced mutagenicity. The decrease in number of MMS-associated His^+^ revertants was significant (*P* < 0.005) in TA102 (73.7%) followed by TA98 (69.0%), TA97a (66.5%), and TA100 (65.3%), as depicted in Tables 7 and 8.

### 3.3. PC and EA Inhibit Microsomal BP-DNA Adducts

**Figure 2:**
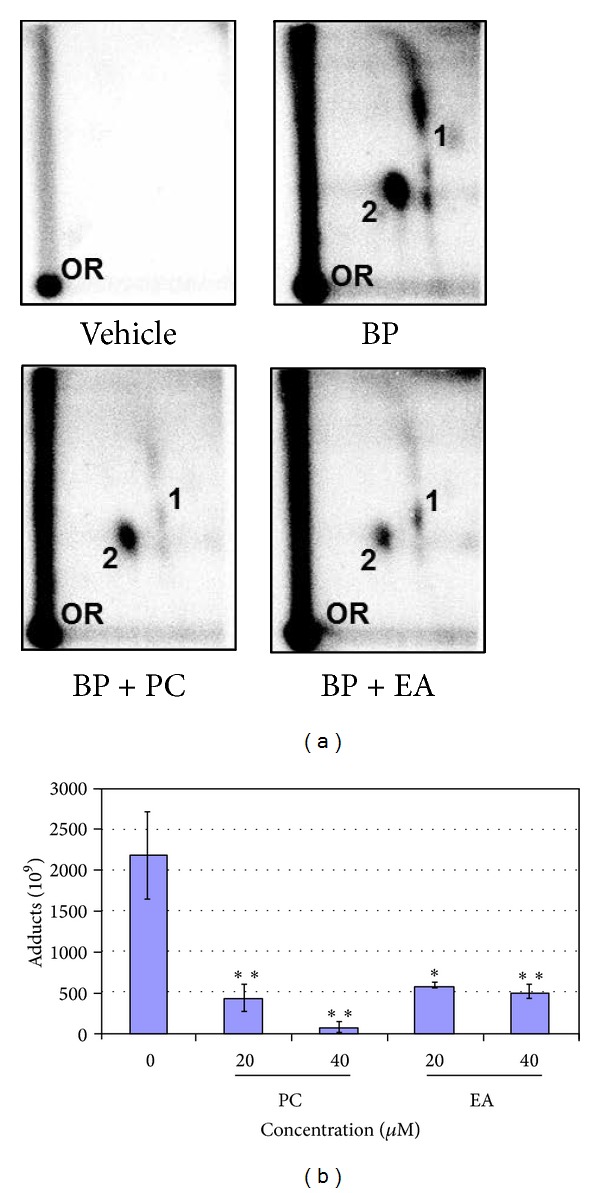
(a) Representative autoradiographs of ^32^P-postlabelling analysis of microsomal-benzo[a]pyrene (BP) DNA adducts in the presence of vehicle alone (2% DMSO), BP (1 *μ*M) + vehicle, BP (1 *μ*M) + punicalagin (PC) (40 *μ*M), and BP (1 *μ*M) + ellagic acid (EA) (40 *μ*M). Adduct 1, anti-benzo[a]pyrene-7,8-diol-9,10-epoxide-dG, and adduct 2, 9-OH-benzo[a]pyrene-4,5-epoxide-dG. PC and EA alone group were not included since we do not expect any background BP-DNA adduct. OR, origin. (b) Inhibition of microsomal BP-induced DNA adducts by PC and EA. DNA adducts were analyzed by ^32^P-postlabeling assay. Data represent an average ± standard error of 4–6 samples. **P < 0.01; *P < 0.05. Part of the figure is reprinted from Mutat. [28] Copyright (2014), with permission from Elsevier.

BP (1 *μ*M) resulted in the formation of two major adducts when incubated with rat liver microsomes in the presence of* st*-DNA (Figure 2). These adducts are products of the interaction of 9-OH-benzo[a]pyrene-4,5-epoxide and dG (adduct 1) and* anti*-BPDE and dG (adduct 2) [31]. No adducts were detected in DNA incubated with vehicle alone (Figure 2(a)). Incubation of* st*-DNA with BP (1 *μ*M), microsomes, and cofactors in the presence of 20 and 40 *μ*M of PC and EA or vehicle produced qualitatively the same DNA adduct profile but the adduct levels were different. Both PC and EA inhibited BP-DNA adducts significantly. As compared with BP alone (219 ± 53 DNA adducts/10^8^ nucleotides; *n* = 6), PC (42.9 ± 16 and 7.2 ± 6.1 DNA adducts/10^8^ nucleotides) and EA (58.6 ± 2.8 and 50.9 ± 8.1 DNA adducts/10^8^ nucleotides) at 20 and 40 *μ*M concentrations, respectively, resulted in significant inhibition of BP-induced DNA adducts (Figure 2(b)).

### 3.4. Antiproliferative Activity of PC and EA

**Figure 3:**
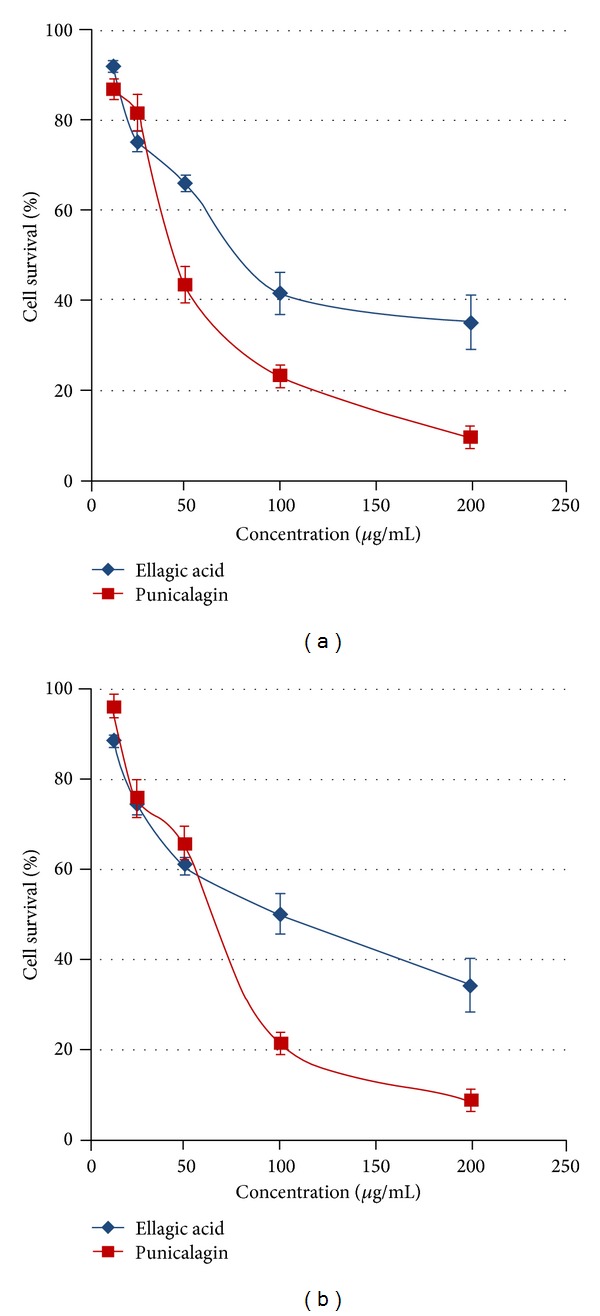
Antiproliferative activity of PC and EA against lung cancer H1299 (a) and A549 (b) cells. Cells were treated with either vehicle or PC and EA at 12.5–200 *μ*g/mL concentrations for 48 h. Data are expressed as percentage of untreated cells, mean ± SD (*n* = 3).

The antiproliferative activities of PC and EA were determined by MTT assay and presented in Figures 3(a) and 3(b). Test compounds showed significant antiproliferative activity against both lung cancer A549 and H1299 cell lines. PC and EA showed dose dependent activity against both of the cell lines after 48 hr. However, the activity of PC was somewhat better than the EA. PC demonstrated significantly high activity and inhibited 57% and 34% of H1299 and A549 cells at 50 *μ*g/mL concentration, respectively. Similarly, at the same concentration (50 *μ*g/mL), EA showed the 34 and 39 percent inhibition against H1299 and A549 lung cancer cell lines, respectively.

## 4. Discussion

Pomegranate (*Punica granatum* L.) fruits are widely consumed fresh and in processed forms as juice, jam, and wine and have been shown to have various protective effects. We have shown that the methanol extract of* Punica granatum* fruit peel possesses antimutagenic, antioxidant, DNA protective, and antiproliferative activity [15, 19]. The husk of pomegranate is rich in ellagitannins (ETs) such as PC, punicalin, gallagic acid, EA, and EA-glycosides [18]. In the present study, PC and one of its hydrolysed products EA were tested for their protective effects against BP-induced DNA adducts and on the genotoxicity induced by various mutagents and promutagens by Ames test. Ames assay serves as a quick and convenient assay to estimate the antimutagenic potential of a compound which is also prescreening of anticancer compounds because standard assays on mice and rats are time-consuming and expensive. Standard mutagens/carcinogens used in this study were well-established mutagens in Ames test.

In our study, none of the tested compounds exhibited any mutagenic effect in the Ames test in the absence of enzymatic metabolism. This suggests that DNA does not seem to be a relevant target for PC and EA, and it did not produce DNA lesions that block DNA synthesis, leading to the induction of the SOS system [32]. A variety of mechanisms can play a vital role in antimutagenic and anticarcinogenic activity of phytocompounds. These mechanisms include inhibition of cell proliferation, signal transduction modulation, scavenging of free radicals, induction of detoxification enzymes, induction of cell-cycle arrest and apoptosis, modulation of cytoskeletal proteins that play a key role in mitosis, and the inhibition of topoisomerase I or II activity [33].

In the Ames test, PC and EA showed antimutagenicity (*P* ≤ 0.05) against mutagens and promutagens in the presence of S9. Since these bacterial strains are unable to metabolize BP and 2AF, to an appreciable extent, a metabolizing system of liver homogenate from Aroclor induced rats (S9) is included in the assay that has been shown to produce a number of reactive intermediates like BP-4,5-oxide and 9-hydroxy-BP-4,5-oxide from BP [34]. The antimutagenic effects of PC and EA could also be due to inhibitory effect against the tested mutagens.

Similarly, the inhibitory activity in the preincubation experiments against 2AF-induced mutagenicity implies that the added modulator interfered with the metabolic activation of the promutagen or tends to interact directly with the ultimate mutagenic metabolite. Cytochrome P-450 enzyme system catalyses the formation of N-hydroxy derivative, that is, N-hydroxy-2-aminoflourene which probably interacts directly with DNA [35]. Thus, the alteration in the structure and function of P-450 enzyme may result in altered rates and differential pathways of metabolism of mutagens and carcinogens and in some cases provide protection against chemically induced mutagenesis. We have recently demonstrated that, besides effect on CYP1A1 and induction of glutathione, PC can inhibit BP by direct inhibition. Moreover, PC upon hydrolysis releases its active metabolite EA, which has been shown to protect DNA by covalently binding [28].

DNA adduct formation represents a net effect of activation and detoxification processes and can be used to determine efficacy of chemopreventive agents. In this study, efficacy of PC and EA was determined by its ability in reducing BP-induced DNA adducts* in vitro*. PC and EA were found to inhibit both anti-BP-7,8-diol-9,10-epoxide-dG and 9-OH-benzo[a]pyrene-4,5-epoxide-dG. However, inhibition of* anti*-BPDE adduct by PC was more pronounced.

Two major pathways can be involved in the inhibition of BP-induced DNA adducts (i) by inhibiting the P450 activity and/or enhancement of phase II enzymes and (ii) by direct conjugation with* anti*-BPDE. It has been previously demonstrated that PC does inhibit* anti*-BPDE-induced DNA adducts and thus ruled out the scavenging of* anti*-BPDE. However, EA has been shown to covalently interact with* anti*-BPDE through its catechol groups as determined by HPLC [36]. Therefore, it is likely that PC is indirectly involved in scavenging* anti*-BPDE through its catechol containing moieties, ellagic acid, and gallic acid. Apparently, the catechol moieties of these constituents were protected in the conjugated PC complex.

This study showed the high antiproliferative activity of PC and EA. Both of the lung cancer cell lines A549 and H1299 showed almost similar level of sensitivity to the tested compounds. The antiproliferative activity of PC and EA against oral, colon, and prostate cancer cell lines has been previously demonstrated [17]. In another study, both PC and EA were found to induce apoptosis via mitochondrial pathway in colon cancer Caco-2 cells but not in normal colon cells. EA arrest the cell cycle in S phase through down-regulation of cyclins A and B1 and up-regulation of cyclin E. It also induces apoptosis via intrinsic pathway through Bcl-XL downregulation, mitochondrial release of cytochrome C, and activation of initiator caspase 9 and effector caspase 3 [37]. Thus, our data corroborates with the literature [17] and clearly demonstrates the antiproliferative potential of PC and EA.

In summary, the data obtained in the present study clearly demonstrated that PC and EA are the major active constituents of pomegranate with promising antimutagenic and protective against DNA damage. Further, these studies demonstrate, for the first time, that both PC and EA possess almost similar level of antimutagenic properties against a variety of mutagens and could be a viable candidate for the future anticancer drugs.

## Supplementary Material

Characteristics of Tester Strains: All the Salmonella typhimurium bacterial strains used in the Ames test carry a defective (mutant) gene that prevents them from synthesizing the essential amino acid histidine. The mutant colonies, which can make histidine are called "revertants". Revertants are identified as colonies that grow in low levels of histidine. Frameshift, transition and base-pair substitution defects are represented to identify the types. The presence of the uvrA/B mutation makes the strains more sensitive to the test articles that induce damage in this manner. The uvrA/B mutation is part of a deletion mutation extending into a gene for biotin synthesis; therefore, the biotin requirement is a result of the deletion of this region. The uvrA/B mutation is indicated by sensitivity to UV light. The rfa mutation changes the properties of the bacterial cell wall and results in the partial loss of the lipopolysaccharide (LPS) barrier increasing permeability of cells to certain types of chemicals. The rfa mutation is indicated by sensitivity to crystal violet. The R factor plasmid (pKM101) makes the strains more responsive to a variety of mutagens. This plasmid carries an ampicillin resistance gene. The pAQ1 plasmid carries a tetracycline resistance gene.

## Figures and Tables

**Table 1:** Effect of punicalagin on the 2-aminofluorene induced mutagenicity in *Salmonella typhimurium*.

Treatment	Dose (*μ*M)	Number of His^+^ revertants colonies/plate (mean ± SE)			
		TA 97a	TA 98	TA 100	TA 102
Spontaneous		144.0 ± 8.7	39.7 ± 3.8	140.3 ± 10.8	325.7 ± 16.6
Positive control (2AF)	1.5 *μ*g	336.7 ± 14.4	254.0 ± 11.8	510.3 ± 23.9	1475.0 ± 38.1
^ a^Punicalagin	50	132.3 ± 8.8	40.7 ± 2.9	136.0 ± 11.7	320.0 ± 21.9
	100	158.0 ± 7.4	36.3 ± 1.8	162.7 ± 15.0	341.0 ± 18.3
	250	165.7 ± 10.9	31.0 ± 1.7	175.3 ± 14.3	356.0 ± 10.4
	500	188.0 ± 9.5	22.3 ± 1.8	191.0 ± 15.0	358.0 ± 19.9
^ b^Punicalagin + 2AF	50	304.7 ± 19.8 (15.7)	210.3 ± 10.7 (20.5)	426.7 ± 28.3 (22.4)	1028.0 ± 21.8*** (38.7)
	100	270.3 ± 10.1* (37.1)	172.3 ± 12.0** (37.5)	358.0 ± 12.2** (43.8)	854.0 ± 31.6*** (54.8)
	250	246.7 ± 12.3** (52.6)	122.0 ± 9.9** (59.2)	304.3 ± 19.5** (61.5)	660.0 ± 24.7*** (72.8)
	500	220.0 ± 9.3*** (78.5)	65.3 ± 4.1*** (81.4)	245.0 ± 9.6*** (83.1)	563.0 ± 29.4*** (81.6)

*R* ^2^		0.98	0.99	0.99	0.99

^a^Negative control; ^b^preincubation test; values in parenthesis are % inhibition of mutagenicity.

*P < 0.05; **P < 0.005 and ***P < 0.001; 2AF: 2-aminofluorene; *R* ^2^: linear regression analysis.

**Table 2:** Effect of punicalagin on the benzo[a]pyrene induced mutagenicity in *Salmonella typhimurium*.

Treatment	Dose (*μ*M)	Number of His^+^ revertants colonies/plate (mean ± SE)			
		TA 97a	TA 98	TA 100	TA 102
Spontaneous		144.0 ± 8.7	39.7 ± 3.8	140.3 ± 10.8	325.7 ± 16.6
Positive control (BP)	1.5 *μ*g	736.7 ± 30.1	165.7 ± 10.2	704.3 ± 27.0	694.0 ± 20.8
Punicalagin	50	132.3 ± 8.8	40.7 ± 2.9	136.0 ± 11.7	320.0±21.9
	100	158.0 ± 7.4	36.3 ± 1.8	162.7 ± 15.0	341.0 ± 18.3
	250	165.7 ± 10.9	31.0 ± 1.7	175.3 ± 14.3	356.0 ± 10.4
	500	188.0 ± 9.5	22.3 ± 1.8	191.0 ± 15.0	358.0 ± 19.9
Punicalagin + BP	50	645.3 ± 28.7 (15.1)	138.0 ± 8.7 (22.1)	530.3 ± 26.4** (30.6)	635.0 ± 23.0 (15.8)
	100	538.3 ± 28.9** (34.3)	105.3 ± 9.0* (46.6)	442.7 ± 31.4** (48.3)	595.0 ± 28.8* (28.0)
	250	431.0 ± 18.4*** (53.5)	65.0 ± 6.8** (74.8)	366.0 ± 20.2*** (64.0)	496.0 ± 17.4** (58.6)
	500	316.0 ± 17.6*** (76.7)	52.7 ± 4.3** (78.8)	270.7 ± 15.6*** (84.5)	438.0 ± 19.5*** (76.2)

*R* ^2^		0.99	0.94	0.99	0.99

^a^Negative control; ^b^preincubation test; values in parenthesis are % inhibition of mutagenicity.

*P < 0.05; **P < 0.005 and ***P < 0.001; BP: benzo[a]pyrene; *R* ^2^: linear regression analysis.

**Table 3:** Effect of punicalagins on the sodium azide induced mutagenicity in *Salmonella typhimurium*.

Treatment	Dose (*μ*M)	Number of His^+^ revertants colonies/plate (mean ± SE)			
		TA 97a	TA 98	TA 100	TA 102
Spontaneous		142.0 ± 5.3	33.7 ± 2.0	128.3 ± 4.9	240.7 ± 7.0
Positive control (NaN_3_)	1.5 *μ*g	256.0 ± 11.7	52.3 ± 1.8	361.3 ± 11.0	370.0 ± 9.0
Punicalagin	50	141.3 ± 5.2	42.3 ± 1.8	189.7 ± 12.0	306.7 ± 13.3
	100	153.7 ± 9.2	35.7 ± 1.8	181.0 ± 7.4	275.3 ± 22.1
	250	158.3 ± 7.1	30.0 ± 1.2	165.3 ± 5.4	250.0 ± 22.9
	500	174.0 ± 6.4	27.3 ± 2.0	187.7 ± 9.8	231.7 ± 24.8
Punicalagin + NaN_3_	50	244.3 ± 6.7 (10.2)	51.0 ± 1.7 (13.3)	342.0 ± 4.7 (11.3)	346.3 ± 23.5 (18.0)
	100	221.0 ± 7.1 (34.2)	47.3 ± 1.8 (30.0)	301.7 ± 7.8* (33.1)	341.0 ± 19.1 (17.6)
	250	202.3 ± 8.7* (54.9)	40.7 ± 2.6* (52.2)	254.3 ± 10.8** (54.6)	310.7 ± 16.0* (42.2)
	500	195.0 ± 10.8* (74.4)	36.0 ± 1.7** (65.3)	232.3 ± 9.4*** (74.3)	381.3 ± 17.6** (59.8)

*R* ^2^		0.99	0.99	0.99	0.91

^a^Negative control; ^b^preincubation test; values in parenthesis are % inhibition of mutagenicity.

*P < 0.05; **P < 0.005 and ***P < 0.001; NaN_3_: sodium azide; *R* ^2^: linear regression analysis.

**Table 4:** Effect of punicalagins on the methyl methanesulfonate induced mutagenicity in *Salmonella typhimurium*.

Treatment	Dose (*μ*M)	Number of His^+^ revertants colonies/plate (mean ± SE)			
		TA 97a	TA 98	TA 100	TA 102
Spontaneous		142.0 ± 5.3	33.7 ± 2.0	128.3 ± 4.9	240.7 ± 7.0
Positive control (MMS)	1.0 *μ*g	449.7 ± 7.2	53.7 ± 1.5	963.0 ± 11.4	1237.0 ± 14.5
Punicalagin	50	141.3 ± 5.2	42.3 ± 1.8	189.7 ± 12.0	292.0 ± 7.7
	100	153.7 ± 9.2	35.7 ± 1.8	181.0 ± 7.4	278.0 ± 12.8
	250	158.3 ± 7.1	30.0 ± 1.2	165.3 ± 5.4	255.0 ± 13.2
	500	174.0 ± 6.4	27.3 ± 2.0	187.7 ± 9.8	254.0 ± 14.3
Punicalagin + MMS	50	418.3 ± 11.7 (10.2)	50.3 ± 1.8 (29.4)	856.3 ± 13.1** (13.8)	1072.0 ± 10.7** (17.5)
	100	390.7 ± 10.2** (19.9)	45.3 ± 2.0* (46.3)	722.0 ± 15.0*** (30.8)	942.0 ± 16.2*** (30.8)
	250	326.0 ± 7.8*** (42.4)	39.7 ± 2.0** (59.2)	601.3 ± 12.3*** (45.3)	738.0 ± 14.8*** (50.8)
	500	251.0 ± 9.0*** (72.1)	35.0 ± 2.1** (70.9)	451.7 ± 15.6*** (66.0)	509.0 ± 19.7*** (74.1)

*R* ^2^		0.95	0.99	0.99	0.99

^a^Negative control; ^b^preincubation test; values in parenthesis are % inhibition of mutagenicity.

*P < 0.05; **P < 0.005 and ***P < 0.001; MMS: methyl methanesulfonate; *R* ^2^: linear regression analysis.

**Table 5:** Effect of ellagic acid on the 2-aminofluorene induced mutagenicity in *Salmonella typhimurium*.

Treatment	Dose (*μ*M)	Number of His^+^ revertants colonies/plate (mean ± SE)			
		TA 97a	TA 98	TA 100	TA 102
Spontaneous		144.0 ± 8.7	39.7 ± 3.8	140.3 ± 10.8	325.7 ± 16.6
Positive control (2AF)	1.5 *μ*g	336.7 ± 14.4	254.0 ± 11.8	510.3 ± 23.9	1475.0 ± 38.1
^ a^Ellagic acid	50	142.3 ± 10.7	50.0 ± 5.1	136.3 ± 12.0	336.0 ± 23.0
	100	159.7 ± 10.5	44.7 ± 3.8	162.7 ± 16.6	275.0 ± 16.8
	250	175.0 ± 15.0	38.3 ± 2.3	178.0 ± 12.7	308.0 ± 17.2
	500	184.0 ± 10.6	32.0 ± 3.6	188.7 ± 12.8	302.0 ± 17.1
^ b^Ellagic acid + 2AF	50	312.3 ± 20.5 (12.5)	214.3 ± 13.9 (19.4)	426.0 ± 18.6* (22.5)	1276.0 ± 38.0* (17.5)
	100	296.7 ± 13.4 (22.6)	171.0 ± 9.5** (39.6)	350.0 ± 12.8** (46.1)	924.0 ± 25.3*** (45.9)
	250	264.0 ± 14.2* (44.9)	132.7 ± 9.3** (56.3)	285.3 ± 13.1** (67.7)	610.0 ± 26.2*** (74.1)
	500	212.0 ± 10.4** (81.7)	76.3 ± 6.1*** (80.0)	240.7 ± 9.8*** (83.8)	431.0 ± 13.6*** (89.0)

*R* ^2^		0.93	0.99	0.99	0.98

^a^Negative control; ^b^preincubation test; values in parenthesis are % inhibition of mutagenicity.

*P < 0.05; **P < 0.005 and ***P < 0.001; 2AF: 2-aminofluorene; *R* ^2^: linear regression analysis.

**Table 6:** Effect of ellagic acid on the benzo[a]pyrene induced mutagenicity in *Salmonella typhimurium*.

Treatment	Dose (*μ*M)	Number of His^+^ revertants colonies/plate (mean ± SE)			
		TA 97a	TA 98	TA 100	TA 102
Spontaneous		144.0 ± 8.7	39.7 ± 3.8	140.3 ± 10.8	325.7 ± 16.6
Positive control (BP)	1.5 *μ*g	736.7 ± 30.1	165.7 ± 10.2	704.3 ± 27.0	694.0 ± 20.8
^ a^Ellagic acid	50	142.3 ± 10.7	50.0 ± 5.1	136.3 ± 12.0	336.0 ± 23.0
	100	159.7 ± 10.5	44.7 ± 3.8	162.7 ± 16.6	275.0 ± 16.8
	250	175.0 ± 15.0	38.3 ± 2.3	178.0 ± 12.7	308.0 ± 17.2
	500	184.0 ± 10.6	32.0 ± 3.6	188.7 ± 12.8	302.0 ± 17.1
^ b^Ellagic acid + BP	50	652.7 ± 35.3 (14.1)	140.0 ± 12.2 (22.2)	580.3 ± 25.6* (21.8)	653.0 ± 34.7 (11.5)
	100	529.0 ± 28.6** (36.0)	107.3 ± 7.9* (48.2)	472.3 ± 19.4** (42.8)	595.0 ± 24.1* (23.6)
	250	333.3 ± 22.2*** (71.8)	86.3 ± 5.8** (62.3)	336.0 ± 17.6*** (70.0)	514.0 ± 21.4** (46.6)
	500	275.3 ± 17.5*** (83.5)	60.7 ± 10.5** (78.6)	245.7 ± 14.4*** (88.9)	366.0 ± 25.0*** (83.7)

*R* ^2^		098	0.97	0.99	0.93

^a^Negative control; ^b^preincubation test; values in parenthesis are % inhibition of mutagenicity.

*P < 0.05; **P < 0.005 and ***P < 0.001; B[a]P: benzo[a]pyrene; *R* ^2^: linear regression analysis.

**Table 7:** Effect of ellagic acid on the sodium azide induced mutagenicity in *Salmonella typhimurium*.

Treatment	Dose (*μ*M)	Number of His^+^ revertants colonies/plate (mean ± SE)			
		TA 97a	TA 98	TA 100	TA 102
Spontaneous		142.0 ± 5.3	33.7 ± 2.0	128.3 ± 4.9	240.7 ± 12.1
Positive control (NaN_3_)	1.5 *μ*g	256.0 ± 11.7	52.3 ± 1.8	361.3 ± 11.0	355.0 ± 15.5
^ a^Ellagic acid	50	189.7 ± 6.4	50.7 ± 1.5	179.7 ± 0.1	310.7 ± 27.0
	100	163.0 ± 5.2	38.0 ± 1.7	170.0 ± 5.7	288.0 ± 21.0
	250	145.3 ± 4.3	34.3 ± 2.4	155.7 ± 10.3	262.3 ± 13.2
	500	134.0 ± 3.8	30.0 ± 1.2	132.3 ± 8.6	248.0 ± 19.1
^ b^Ellagic acid + NaN_3_	50	242.0 ± 11.8 (21.1)	52.0 ± 1.7 (20.0)	340.7 ± 9.4 (11.4)	345.3 ± 26.7 (21.9)
	100	219.3 ± 7.1 (39.4)	48.3 ± 2.7 (27.9)	312.3 ± 8.4* (25.6)	328.0 ± 28.2 (40.3)
	250	196.7 ± 8.2* (53.6)	43.3 ± 2.6* (50.0)	266.0 ± 7.8** (46.4)	308.0 ± 23.6* (50.7)
	500	168.0 ± 7.0** (72.1)	38.0 ± 1.7** (64.2)	210.3 ± 12.4*** (65.9)	288.3 ± 19.2** (62.3)

*R* ^2^		0.99	0.98	0.99	0.97

^a^Negative control; ^b^preincubation test; values in parenthesis are % inhibition of mutagenicity.

*P < 0.05; **P < 0.005 and ***P < 0.001; NaN_3_: sodium azide; *R* ^2^: linear regression analysis.

**Table 8:** Effect of ellagic acid on the methyl methanesulfonate induced mutagenicity in *Salmonella typhimurium*.

Treatment	Dose (*μ*M)	Number of His^+^ revertants colonies/plate (mean ± SE)			
		TA 97a	TA 98	TA 100	TA 102
Spontaneous		142.0 ± 5.3	33.7 ± 2.0	128.3 ± 4.9	240.7 ± 7.0
Positive control (MMS)	1.5 *μ*g	449.7 ± 7.2	53.7 ± 1.5	963.0 ± 11.4	1237.0 ± 14.5
^ a^Ellagic acid	50	189.7 ± 6.4	50.7 ± 1.5	179.7 ± 9.1	338.0 ± 15.6
	100	163.0 ± 5.2	38.0 ± 1.7	170.0 ± 5.7	311.0 ± 12.1
	250	145.3 ± 4.3	34.3 ± 2.4	155.7 ± 10.3	274.0 ± 7.6
	500	134.0 ± 3.8	30.0 ± 1.2	132.3 ± 8.6	228.0 ± 11.0
^ b^Ellagic acid + MMS	50	426.3 ± 11.6 (9.0)	53.0 ± 1.2 (22.2)	900.3 ± 16.3* (8.0)	1071.0 ± 16.7** (18.5)
	100	378.0 ± 10.8** (25.0)	48.3 ± 2.0 (34.0)	754.0 ± 8.2*** (26.4)	956.0 ± 19.6*** (30.3)
	250	321.3 ± 10.8*** (42.2)	44.0 ± 2.1* (50.0)	598.7 ± 14.2*** (45.1)	810.0 ± 19.1*** (44.3)
	500	239.7 ± 10.8*** (66.5)	37.3 ± 2.6** (69.0)	420.3 ± 6.9*** (65.3)	506.0 ± 13.5*** (72.4)

*R* ^2^		0.98	0.99	0.99	0.95

^a^Negative control; ^b^preincubation test; values in parenthesis are % inhibition of mutagenicity.

*P < 0.05; **P < 0.005 and ***P < 0.001; MMS: methyl methanesulfonate; *R* ^2^: linear regression analysis.
