# Supplementary material for: Punicalagin and Ellagic Acid Demonstrate Antimutagenic Activity and Inhibition of Benzo[a]pyrene Induced DNA Adducts
Source: Biomed Res Int. 2014 May 14;2014:467465. doi: 10.1155/2014/467465 (PMC4052943; doi:10.1155/2014/467465)
Supplement: Supplementary file 1 — Characteristics of Tester Strains: All the Salmonella typhimurium bacterial strains used in the Ames test carry a defective (mutant) gene that prevents them from synthesizing the essential amino acid histidine. The mutant colonies, which can make histidine are called "revertants". Revertants are identified as colonies that grow in low levels of histidine. Frameshift, transition and base-pair substitution defects are represented to identify the types. The presence of the uvrA/B mutation makes the strains more sensitive to the test articles that induce damage in this manner. The uvrA/B mutation is part of a deletion mutation extending into a gene for biotin synthesis; therefore, the biotin requirement is a result of the deletion of this region. The uvrA/B mutation is indicated by sensitivity to UV light. The rfa mutation changes the properties of the bacterial cell wall and results in the partial loss of the lipopolysaccharide (LPS) barrier increasing permeability of cells to certain types of chemicals. The rfa mutation is indicated by sensitivity to crystal violet. The R factor plasmid (pKM101) makes the strains more responsive to a variety of mutagens. This plasmid carries an ampicillin resistance gene. The pAQ1 plasmid carries a tetracycline resistance gene. [file 467465.f1.docx]

**Punicalagin and Ellagic Acid Demonstrates Antimutagenic Activity and Inhibition of Benzo[a]pyrene Induced DNA Adducts**

Maryam Zahin^1*^, Iqbal Ahmad^1^, Ramesh Gupta^2,4^ and Farrukh Aqil^2, 3, 5^

**Supplementary Materials**

**Table S1.** Characteristics of *Salmonella typhimurium* tester strains.

| Strains | Genetic markers | | | | Plasmids | Mutational events |
| --- | --- | --- | --- | --- | --- | --- |
|  | Gene effected | DNA repair mutation | LPS | Biotin requirement |  |  |
| TA 97a | hisD | uvrB | rfa | bio- | pkM 101 | frameshift |
| TA 98 | hisD | uvrB | rfa | bio- | pkM 101 | frameshift |
| TA 100 | hisG | uvrB | rfa | bio | pkM 101 | base-pair substitution |
| TA 102 | hisG | - | rfa | - | pkM 101 & pAQ1 | transition |
| TA 104 | hisG | uvrB | rfa | bio | pkM 101 | transition |
